# Prognostic value of serum CYFRA 21-1 1 in patients with anal canal squamous cell carcinoma treated with radio(chemo)therapy

**DOI:** 10.1186/s12885-018-4335-4

**Published:** 2018-04-13

**Authors:** Mathieu Gauthé, Marion Richard-Molard, Eugénie Rigault, Bruno Buecher, Pascale Mariani, Dominique Bellet, Wulfran Cacheux, Astrid Lièvre

**Affiliations:** 1 Médecine nucléaire, Institut Curie, Hôpital Paris, 26 rue d’Ulm, 75005 Paris, France; 2 Médecine nucléaire, Hôpital Tenon, Assistance Publique-Hôpitaux de Paris, 4 rue de la Chine, 75020 Paris, France; 3 Université Pierre et Marie Curie, 4 Place Jussieu, Paris, France; 4 Département de radiothérapie, Institut Curie, Hôpital René Huguenin, 35 rue Dailly, 92210 Saint-Cloud, France; 5 Service des maladies de l’appareil digestif, Centre Hospitalier Universitaire de Rennes, 2 rue Henri Le Guilloux, Rennes, 35033 France; 6 Faculté de Médecine, Université de Rennes 1, 2 avenue du Pr. Léon Bernard, 35043 Rennes, France; 7 INSERM U1242, COSS (chemistry, oncogenesis, stress and signaling), , Rue Bataille Flandres-Dunkerque, 35043 Rennes, France; 8 Département d’oncologie médicale, Institut Curie, Hôpital Paris, 26 rue d’Ulm, 75005 Paris, France; 9 Département de chirurgie oncologique, Institut Curie, Hôpital Paris, 26 rue d’Ulm, 75005 Paris, France; 10 Laboratoire d’oncobiologie, Département de biopathologie, Institut Curie, Hôpital René Huguenin, 35 rue Dailly, 92210 Saint-Cloud, France; 11 Département d’oncologie médicale, Institut Curie, Hôpital René Huguenin, 35 rue Dailly, 92210 Saint-Cloud, France; 12 Unité de pharmacogénomique, département de génétique, Institut Curie, Hôpital Paris, 26 rue d’Ulm, 75005 Paris, France

**Keywords:** CYFRA 21-1, Anal carcinoma, Tumour marker, Recurrence, Prognosis

## Abstract

**Background:**

We aimed to assess the prognostic value of CYFRA 21-1 in a series of patients with anal canal squamous cell carcinoma treated by radiation-based therapy.

**Methods:**

All patients with anal cancer referred between September 2005 and July 2013 were considered. Patients with diagnosis of anal squamous cell carcinoma and in whom pre- and post-treatment serum CYFRA 21-1 levels were available were included. Serum CYFRA 21-1 levels at initial workup and after therapy were collected. Survival rates were estimated using the Kaplan–Meier method. Cox regression analysis was used to evaluate prognostic variables for prediction of outcomes.

**Results:**

Eighty-two patients were included. Median follow-up was 60 months (range: 8–128). Pre-treatment serum CYFRA 21-1 levels were significantly correlated with tumour stage (*p* < 0.001). Normal post-treatment serum CYFRA 21-1 level was significantly correlated with tumour complete response (*p* = 0.004). Elevated post-treatment serum CYFRA 21-1 level was significantly associated with poorer progression-free survival (*p* = 0.02) and overall survival (*p* = 0.003). T stage and post-treatment serum CYFRA 21-1 were independent prognostic factors for overall survival (*p* = 0.04 and 0.03, respectively).

**Conclusions:**

Serum CYFRA 21-1 appears to be a useful marker for the monitoring of anal squamous cell carcinoma patients. Elevated post-treatment value appears to be correlated with treatment failure.

## Background

Anal canal squamous cell carcinoma (ASCC) is a rare but increasingly common malignancy, accounting for less than 1% of all malignancies and cancer deaths [19]. The majority of ASCCs are diagnosed at a localized or locally advanced stage, with a 5-year survival of 65% [6, 19]. Concomitant chemoradiotherapy (CRT) is the standard of care for locally advanced tumours, with a complete response rate of more than 80% [1]. Salvage abdominoperineal resection (APR) is the standard treatment for local failure or recurrence after radiation-based therapy, which is observed in more than 30% of patients [5, 10].

Tumour size and lymph node involvement are the two major prognostic factors reported and used in clinical practice to guide treatment of ASCC patients [2]. Better selection of patients at high risk of failure of radiation-based therapy and new pre-treatment prognostic factors are required in this setting. Squamous cell carcinoma antigen (SCC Ag) has recently been reported to be a potentially useful serum prognostic marker in ASCC patients [25]. Cytokeratin 19 fragment 21–1 (CYFRA 21-1) is a soluble fragment of cytokeratin 19. Elevated levels of serum CYFRA 21-1 have been described in various malignancies, such as lung [13] and breast [18] cancers, but its usefulness in ASCC has not yet been evaluated.

In our department, serum CYFRA 21-1 assay has been routinely performed at the initial diagnostic workup and after radiation-based therapy since 2005.

The aim of this study was to assess the prognostic value of CYFRA 21-1 in a series of patients with ASCC treated by radiotherapy (RT) or CRT.

## Methods

### Study population

All patients with ASCC referred to one of the two hospitals of our institution between September 2005 and July 2013 were retrospectively reviewed. Inclusion criteria were patients with a recent histologically proven diagnosis of ASCC, treated with exclusive RT or CRT and for whom serum CYFRA 21-1 samples were available before and after treatment. Exclusion criteria were other histological types of anal canal tumours and patients with distant metastases (stage IV). Patients were staged according to the AJCC classification [6]. Treatment was decided during a multidisciplinary meeting.

### Tumour markers assays

Serum CYFRA 21-1 levels were measured with a Time-Resolved Cryptate Emission assay (BRAHMS CYFRA 21-1 Kryptor, Thermo Scientific) according to the manufacturer’s instructions and were determined at initial diagnostic workup and after radiation-based therapy. The upper limit of normal values with this assay is 1 ng/ml. Pre-treatment CYFRA 21-1 level was measured in the month before the start of radiation-based therapy. For the study, we considered post-treatment CYFRA 21-1 as the assay performed between 2 and 6 months after the end of radiation-based therapy, which is the optimal time to assess clinical response to treatment. When two assays were available during this period for a patient, only the latest one was considered for the analysis. Serial serum CYFRA 21-1 levels after 6 months during surveillance were not analysed in this study as CYFRA 21-1 assays were not systematically and uniformly performed in all patients.

### Treatment

Patients were treated according to national guidelines published by the *Thésaurus national de cancérologie digestive* (French Digestive Oncology Thesaurus) [14]. Stage I and low-risk stage II (T2 < 3 cm - N0) cancers were treated by exclusive external RT with a total dose of 45 Gy plus a boost dose of 20 Gy to the primary target volume. High-risk stage II (T2 > 3 cm and T3 - N0) and stage III (A and B) cancers were treated by concurrent CRT, comprising pelvic external radiotherapy (total dose of 45 Gy plus a boost dose of 20 Gy to the primary target volume) associated with concomitant 5-fluorouracil (5FU) – cisplatin or 5FU - mitomycin C chemotherapy.

### Patient follow-up

Patients were followed at least weekly during treatment. After completing therapy, they were evaluated clinically every 3 to 4 months for 2 years, then every 6 months for 3 years, and annually thereafter. Contrast-enhanced CT scan of the chest, abdomen and pelvis was performed annually [14]. Complete remission was defined as complete resolution of palpable tumour on clinical examination and/or imaging at the censor date. Persistent disease was defined as histologically proven residual disease within 6 months after completion of treatment. Recurrence was defined as reappearance of progressive disease on clinical examination and/or imaging after a complete response to RT/CRT before the censor date. Additional imaging and laboratory tests were performed in case of suspected recurrence. Residual or recurrent disease was confirmed by biopsies when other assessments were ambiguous.

### Statistical analysis

Data were analyzed using SPSS statistics (IBM Corporation). The Kruskal–Wallis one-way analysis was used for multiple comparisons between pre-treatment and post-treatment serum CYFRA 21-1 of the AJCC 2010 staging groups I to IIIB and according to disease outcome groups (complete remission, partial response and recurrence). The Mann-Whitney U-test was used for comparisons between pairs of independent variables classified according to AJCC 2010 staging. The Chi^2^ test (Fisher’s exact method) was used for comparisons between variables. Survival rates were estimated using the Kaplan–Meier method. PFS was calculated as the interval from the date of initiation of RT/CRT to the date of recurrence, progressive disease or death from any cause. OS was calculated from the date of initiation of RT/CRT to the date of death from any cause or last follow-up. Patients who did not present an event of interest were censored at the time of last follow-up. Censoring was performed 60 months (5 years) after the end of RT/CRT. Time-dependent receiver-operating-characteristic (ROC) analysis was used to determine area under the curve (AUC) and cut-off to estimate the prognostic ability of CYFRA 21-1. Serum CYFRA 21-1 values were categorized as normal or elevated according to this cut-off, and considered as such for survival analyses. Survival curves were compared using the log-rank test. Cox regression univariate analysis was used to evaluate prognostic variables for prediction of PFS and OS. Cox regression multivariate analysis was performed to determine the association of risk factors identified on univariate analysis (*p* < 0.05) adjusted for confounders. The prognostic factors analyzed included pre-treatment and post-treatment serum CYFRA 21-1, age at diagnosis, gender, T (tumour) stage, N (node) stage, and tumour stage (I/II/IIIA/IIIB). The proportional hazard assumption was checked by using Schoenfeld residuals for each covariable. A *p* value less than 0.05 was considered to be statistically significant.

## Results

### Patient, tumour and treatment characteristics

Between September 2005 and July 2013, serum CYFRA 21-1 assay was performed before and after RT/CRT in 82 patients. Patient characteristics are summarized in Table 1.

**Table 1:** Patient, tumour and treatment characteristics

Patient characteristics	No. of patients (%)
Total	82
Sex (male/female)	8 (9.8%) / 74 (90.2%)
Median age	63.5 ± 11.9 years (range: 40–96 years)
Positive HIV serology	1 (1.2%)
TNM stage	
T1	6 (7.3%)
T2	34 (41.5%)
T3	27 (32.9%)
T4	15 (18.3%)
N0	34 (41.5%)
N1	20 (24.4%)
N2	13 (15.9%)
N3	15 (18.3%)
Radiotherapy and chemotherapy	
Exclusive radiotherapy	22 (26.8%)
Concurrent CRT	60 (73.2%)
Cisplatin-based	50
Mitomycin-C-based	6
Capecitabine	4

Most patients were females (90.2%) and one patient (1.2%) was HIV positive. Human papilloma virus (HPV) status was available in only 12/82 patients (14.6%). All tested patients were positive for HPV16. According to the AJCC classification, 4 patients were stage I (4.9%), 25 were stage II (30.5%), 23 were stage IIIA (28%) and 30 were stage IIIB (36.6%). Twenty-two patients were treated by exclusive RT (median radiation dose to the primary tumour: 60.5 ± 4.7 Gy). The other 60 patients were treated by concurrent CRT, combining external RT (median radiation dose to the primary tumour: 64.7 ± 6.2 Gy) and chemotherapy, mostly with a platinum-based regimen (*n* = 50) (Table 1).

### Follow-up

Median follow-up was 60 months (range: 8–128). Patients’ follow-up is summarized in Table 2. Five patients (6.1%) did not achieve complete response to RT or CRT, with histological proof of persistent disease. Fifteen patients (18.3%) presented tumour recurrence, including 12 histologically documented recurrences. Nine patients experienced loco-regional recurrence and were treated by APR. One patient developed an isolated liver metastasis treated by surgery and 5 patients developed an unresectable distant metastatic recurrence. Twenty deaths occurred: 15 due to ASCC progression and 5 due to a non-squamous cell carcinoma (2 lung, 2 colon and 1 oesophageal cancers). One patient was lost to follow-up after 19 months. The five-year PFS and OS rates were 75.6% and 76.7% respectively (100% and 100% for stage I, 76 and 88% for stage II, 78.3% and 69.6% for stage IIIA and 70% and 76.7% for stage IIIB ASCC).

**Table 2:** Patient’s follow-up

Patient characteristics	No. of patients (%)
Number	82
Median follow-up	60 months (range: 8–128)
Incomplete response to RT or CRT	5 (6.1%)
Recurrence	15 (18.3%)
Resectable loco-regional recurrence	9
Unresectable metastatic recurrence	5
Resectable metastatic recurrence	1
Death	20 (24.4%)
In relation with anal cancer	15
In relation with another cause	5

### Time-course of serum CYFRA 21-1 levels

#### Pre-treatment and post-treatment serum marker levels according to tumour stage

**Fig. 1:**
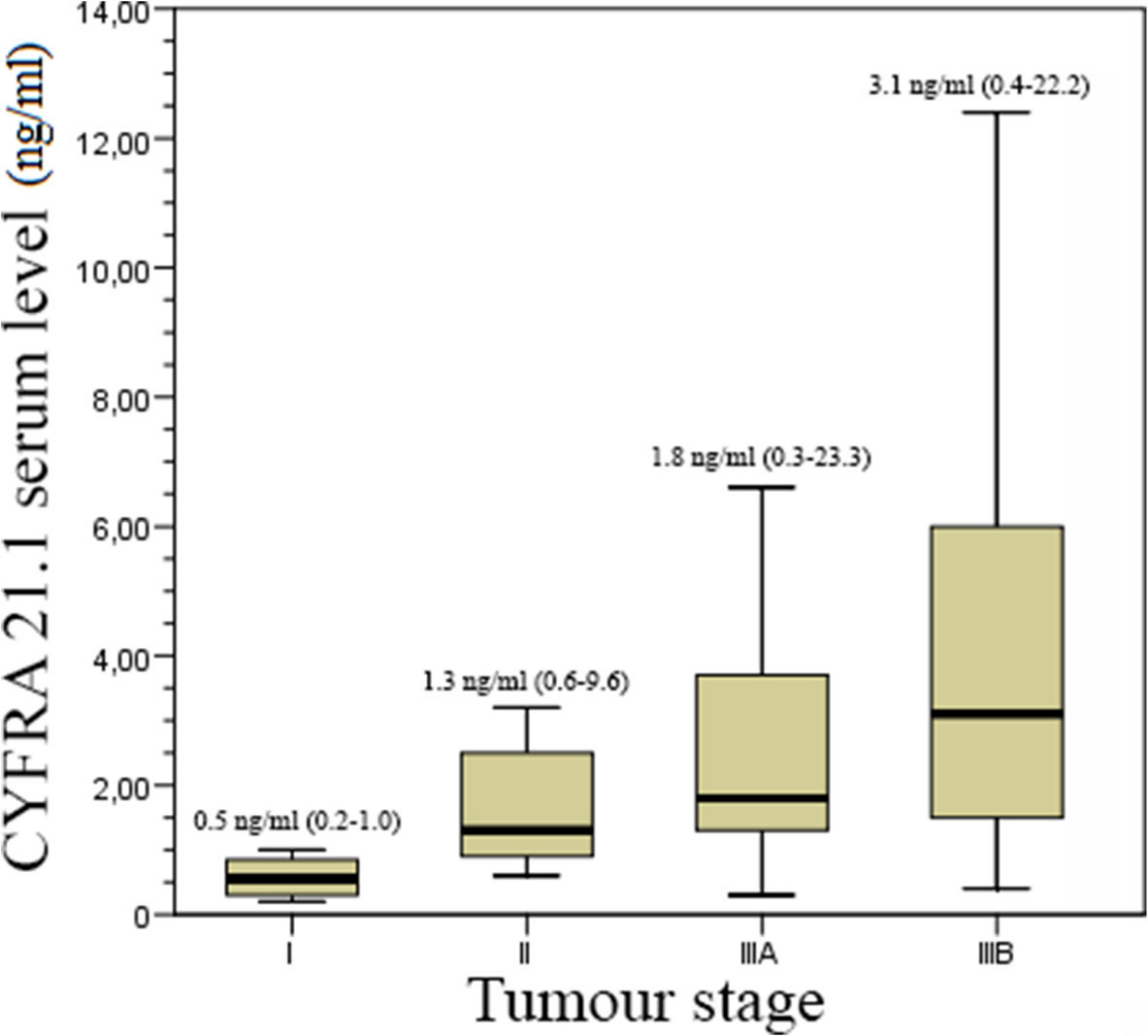
Box plot representing pre-treatment serum CYFRA 21-1 levels (*n* ≤ 1 ng/ml) according to tumour stage. Median and range are noted above each box

Pre-treatment serum CYFRA 21-1 levels for all 82 patients are shown in Fig. 1. The mean time to serum CYFRA 21-1 assay after the end of RT/CRT was 3.8 ± 1.6 months. Pre-treatment serum CYFRA 21-1 levels were significantly correlated with tumour stage (*p* < 0.001; Chi^2^ test), while post-treatment serum CYFRA 21-1 levels were not (median values and ranges: 0.5 ± 0.2 ng/ml, 0.8 ± 0.4 ng/ml, 1 ± 0.5 ng/ml and 0.9 ± 0.7 ng/ml for stages I, II, IIIA and IIIB respectively; *p* = 0.23; Chi^2^ test). Using the Mann-Whitney U-test to compare differences between serum CYFRA 21-1 levels in the two groups according to tumour stage, pre-treatment serum CYFRA 21-1 levels were significantly different between all pairs of groups (I vs. II-III and I-II vs. III) (*p* = 0.001). In contrast, post-treatment serum CYFRA 21-1 levels were not significantly different between of the compared groups.

#### Serum marker levels according to treatment response and recurrence

Normal post-treatment serum CYFRA 21-1 level was significantly more frequent in patients with tumour complete response (*p* = 0.004) (Table 3). A trend was also observed towards more frequently elevated post-treatment serum CYFRA 21-1 in patients with overall failure to treatment (non-responders and patients who relapsed) (*p* = 0.06).

**Table 3:** Pre-treatment and post-treatment serum CYFRA 21-1 levels according initial response to radiation-based therapy and long term treatment failure (defined as partial response or recurrence after complete response to initial radiation-based therapy)

Initial response to radiation-based therapy	Complete response	Partial response	Chi^2^ (p)
n (%)	77 (93.9)	5 (6.1)	
Pre-treatment			
Normal (≤ 1 ng/ml)	22 (28.6)	0 (0)	
Elevated (> 1 ng/ml)	55 (71.4)	5 (100)	1.95 (*p* = 0.32)
Post-treatment			
Normal (≤ 1 ng/ml)	53 (71)	0 (0)	
Elevated (> 1 ng/ml)	24 (29)	5 (100)	9.73 (*p* = 0.004)
Long term treatment failure	No	Yes	Chi^2^ (p)
n (%)	62 (75.6)	20 (24.4)	
Pre-treatment			
Normal (≤ 1 ng/ml)	19 (30.6)	3 (15)	
Elevated (> 1 ng/ml)	43 (69.4)	17 (85)	1.89 (*p* = 0.25)
Post-treatment			
Normal (≤ 1 ng/ml)	44 (71)	9 (45)	
Elevated (> 1 ng/ml)	18 (29)	11 (55)	4.46 (*p* = 0.06)

Among the 15 patients who presented a recurrence after a complete response to RT or CRT, 6 had an elevated post-treatment CYFRA 21-1 level. In these patients, the median delay between post-treatment CYFRA 21-1 assay and recurrence was 6.6 months (range: 2.5–38.9 months).

### Prognostic value of CYFRA 21-1 levels and other prognostic factors

**Fig. 2:**
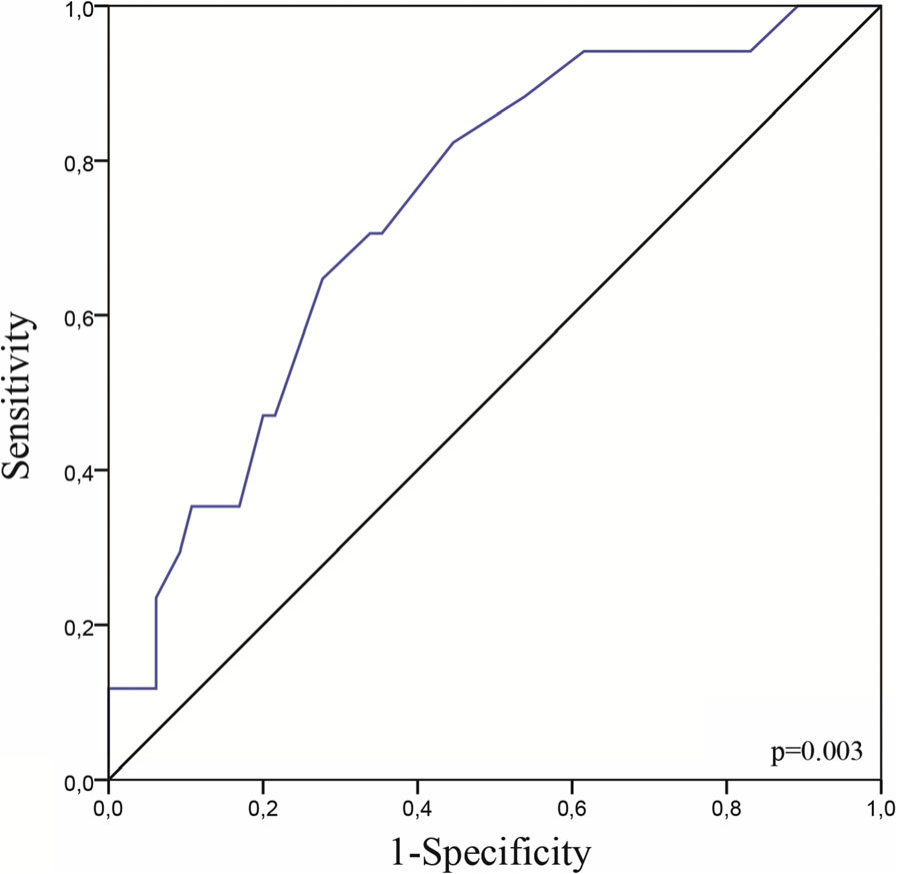
Receiver operating characteristic curve for post-treatment serum CYFRA 21-1. Area under the curve = 0.74 [95% confidence interval: 0.61–0.86], *p* = 0.03

From the ROC analysis (Fig. 2), the post-treatment serum CYFRA 21-1 cut-off value was 0.97 ng/ml (AUC = 0.74; *p* = 0.003). Using this cut-off, sensitivity, specificity, predictive positive and negative values were calculated at 71%, 66%, 68% and 69% respectively. Thus, we chose the value of 1 ng/ml as cut-off for our analyses, because it was close to 0.97 ng/ml and also correspond to the manufacter’s threshold for defining normality.

**Fig. 3:**
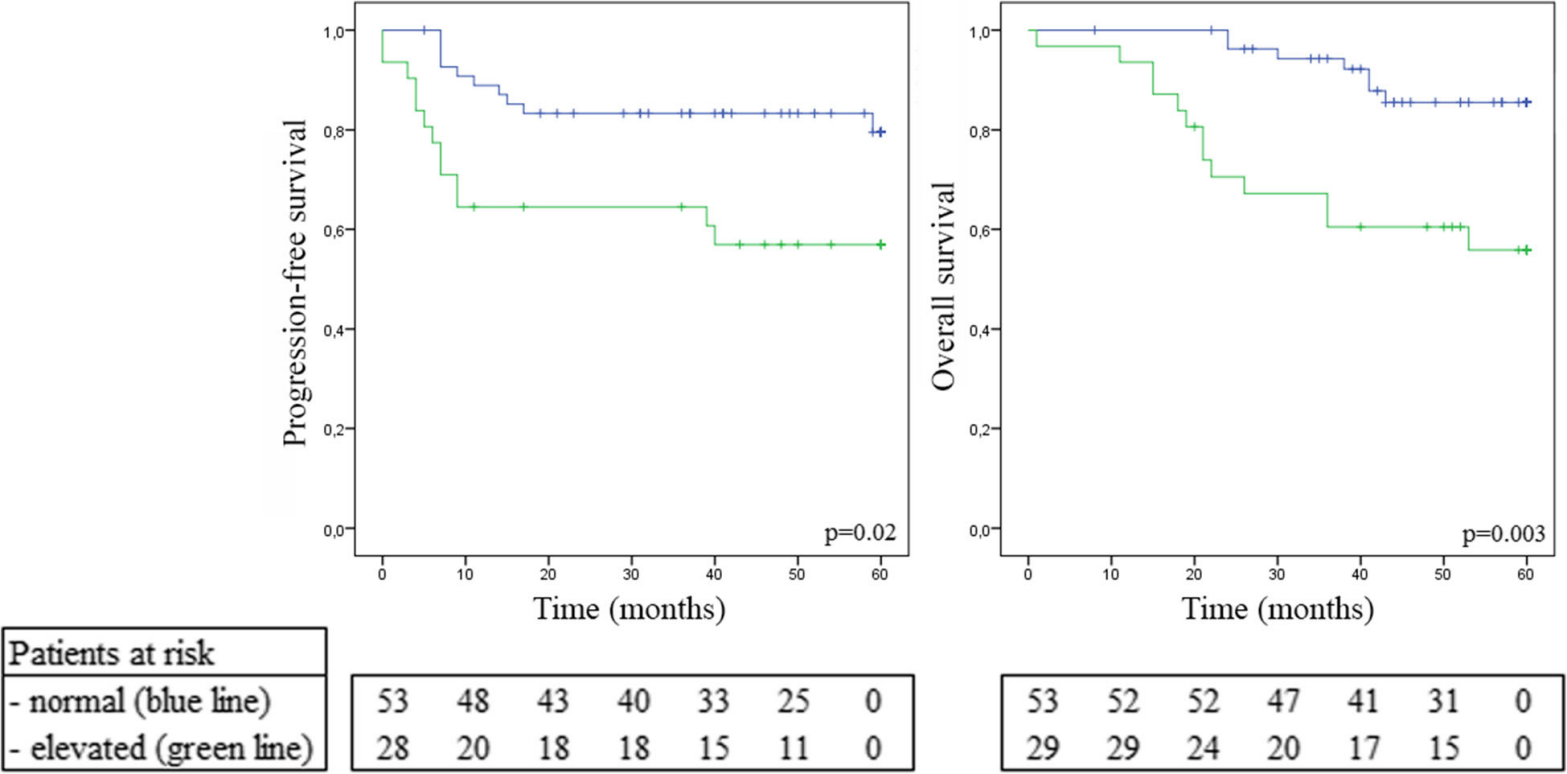
Survival according to elevated post-treatment serum CYFRA 21.1 levels. Green line indicates patients with elevated post-treatment serum CYFRA 21.1. Blue line indicates patients with normal post-treatment serum CYFRA 21.1 (n ≤ 1 ng/ml). Bars indicate censored individuals

On Kaplan-Meier analysis, elevated post-treatment serum CYFRA 21-1 level was significantly associated with poorer PFS and OS (*p* = 0.02 and 0.003, respectively) (Fig. 3). A significant difference in OS was also observed according to pre-treatment serum CYFRA 21-1 levels (*p* < 0.05). No statistically significant difference was observed for PFS according to pre-treatment serum CYFRA 21-1 levels (*p* = 0.2).

In Cox univariate analysis, elevated post-treatment serum CYFRA 21-1 and advanced T stage were significant predictors of outcome for PFS and OS (*p* = 0.03 and 0.04 for PFS; *p* = 0.006 and 0.007 for OS, respectively) (Tables 4 and 5).

**Table 4:** Cox univariate analysis for progression-free survival, with a time dependency added to CYFRA 21-1 pre-treatment and post-treatment levels

	Univariate analysis	
	HR [95%CI]	*p*
Age at diagnosis (> vs. ≤ 65 years)	0.95 [0.39;2.33]	0.91
Gender (male/female)	0.57 [0.17;1.96]	0.47
T stage (T3/T4 vs. T1/T2)	2.65 [1.06;6.34]	0.04
Nodal status (positive/negative)	1.07 [0.44;2.62]	0.88
Clinical stage (IIIA/IIIB vs. I/II)	1.36 [0.52;3.54]	0.53
Chemotherapy associated with radiation therapy (yes/no)	1.02 [0.37;2.8]	0.97
CYFRA 21-1 levels (elevated /normal)		
Pre-treatment	2.17 [0.64;7.42]	0.22
Post-treatment	2.64 [1.09;6.36]	0.03

**Table 5:** Cox univariate analysis for overall survival, with a time dependency added to CYFRA 21-1 pre-treatment and post-treatment levels

	Univariate analysis	
	HR [95%CI]	*p*
Age at diagnosis (> vs. ≤ 65 years)	2.13 [0.81;5.61]	0.12
Gender (male/female)	0.52 [0.16;3.07]	0.30
T stage (T3/T4 vs. T1/T2)	5.44 [1.36;16.48]	0.007
Nodal status (positive/negative)	1.34 [0.49;3.63]	0.56
Clinical stage (IIIA/IIIB vs. I/II)	2.72 [0.78;9.46]	0.12
Chemotherapy associated with radiation therapy (yes/no)	0.79 [0.28;2.27]	0.67
CYFRA 21-1 levels (elevated /normal)		
Pre-treatment	5.99 [0.80;45.22]	0.08
Post-treatment	4.05 [1.49;10.95]	0.006

In Cox multivariate analysis, T stage and post-treatment serum CYFRA 21-1 were independent prognostic factors for OS only (*p* = 0.04 and 0.03, respectively), but not for PFS.

## Discussion

The clinical value of cytokeratins as tumour markers has already been reported for diagnostic or prognostic purposes and for evaluation of treatment efficacy, especially in non-small-cell lung cancers (NSCLC) and breast cancers [3, 12, 17, 18]. Although it has recently been shown to be clinically useful in various types of squamous cell carcinoma [4, 9, 20, 26], very little is known about the value of serum CYFRA 21-1 in ASCC [8]. CYFRA 21-1 is a soluble fragment of cytokeratin 19 and tumours arising within the anal canal are most often keratinizing tumours [21, 22], suggesting the possible value of this marker in ASCC. Some studies have suggested that CYFRA 21-1 could be superior to SCC Ag in predicting response to therapy (chemotherapy or CRT) or survival in NSCLC and SCC of the oesophagus and the cervix [15, 20, 24], while another study has reported that both tumour markers could serve as useful markers to assess response to therapy and predict tumour recurrence in head and neck SCC [4]. CYFRA 21-1 is routinely used in our centre, in preference to other serum markers, due to its high specificity in the follow-up of ASCC patients [8].

The results of the present study demonstrate, for the first time, that pre-treatment serum CYFRA 21-1 levels are significantly correlated with ASCC tumour stage, as previously reported in other cancers [4, 20, 26]. However, this result does not mean that serum CYFRA 21-1 levels could constitute a potential diagnostic biomarker for ASCC because the sensitivity of this marker and the levels observed in patients with ASCC compared to healthy patients are unknown. Elevated serum CYFRA 21-1 levels are rare in the healthy population, but have been described in up to one-third of patients with cirrhosis, renal failure, or infectious lung disease [13, 16]. None of the patients included in our study had a diagnosis of these 3 conditions.

Several previous studies have also demonstrated the prognostic value of pre-treatment serum CYFRA 21-1 in various types of cancer [20, 27] and Williams et al. recently demonstrated a potential prognostic value of pre-treatment serum SCC Ag levels in a larger cohort of ASCC patients who received concurrent CRT [25]. Our study did not find a prognostic value of pre-treatment serum CYFRA 21-1 in ASCC, possibly because of the small sample size of this series.

In our study, we chose to consider serum CYFRA 21-1 values as a binary parameter, elevated or not compared to a defined cut-off, as this is more relevant in routine practice than using absolute values. Thus, by using this method, an elevated post-treatment serum CYFRA 21-1 level was an independent factor of poor prognosis in terms of PFS and OS. It was not possible to analyse whether patients with very elevated post-treatment CYFRA-21 level had a poorest prognosis as only 2 patients in our series had a serum marker level > 3 ng/ml (one presented a recurrence). In our series, elevated post-treatment serum CYFRA 21-1 level also tended to be associated with treatment failure. In patients who relapsed after a complete response to RT or CRT, the median time between elevated post-treatment CYFRA 21-1 level and clinico-radiological recurrence was 6.6 months (range: 2.5–38.9 months). Considered together these results suggest that post-treatment serum CYFRA 21-1 level could have a prognostic impact and be a valuable marker of treatment efficacy that could be used to detect recurrence earlier than clinical and/or radiological data. Therefore, it could be a useful tool in patient monitoring to optimize the follow-up and guide the decision to perform salvage APR after RT/CRT [11, 23].

The main limitations of our study are its retrospective design and the long inclusion period due to the rarity of ASCC. This study failed to demonstrate a statistically correlation between survival and N stage, which could be attributed to lack of power due to the small sample size of our population and the imbalance between clinical stages (stage I was considerably under-represented). Another limitation of our study remains the low specificity of the post-treatment CYFRA 21-1 level. Finally, it is noteworthy that the prognostic value of elevated serum CYFRA 21-1 observed in our study was obtained with a cut-off value of 1 ng/ml, which corresponds to the cut-off recommended by the manufacturer. However, some studies assessing this tumour marker in other malignancies have reported the prognostic value of higher cut-offs [7, 18].

In view of these limitations, further prospective studies on independent series of larger sample size are needed to determine if serum CYFRA 21-1 levels has a clinical utility as a prognostic and predictive marker in ASCC patients.

Further analyses are also required to determine the optimal interval after exclusive RT/CRT for post-treatment CYFRA 21-1 assay and to assess the value of serum CYFRA 21-1 kinetics during the follow-up for the diagnosis of recurrence after treatment.

## Conclusion

Serum CYFRA 21-1, especially post-treatment levels, appears to be a useful prognostic and therapeutic marker for the monitoring of ASCC patients treated by exclusive RT/CRT. An abnormally elevated value after exclusive RT/CRT appears to be correlated with treatment failure, which should prompt proposal of closer patient follow-up and possibly early salvage APR.

Further large-scale prospective studies should therefore be conducted on this serum biomarker in ASCC patients.

## Abbreviations

5FU: 5-fluorouracil
APR: Abdominoperineal resection
ASCC: Anal canal squamous cell carcinoma
AUC: Area under the curve
CRT: Concomitant chemoradiotherapy
CT: Computerized tomography
CYFRA 21-1: Cytokeratin 19 fragment 21–1
NSCLC: Non-small-cell lung cancers
OS: Overall survival
PFS: Progression-free survival
ROC: Receiver-operating-characteristic
RT: Radiotherapy
SCC Ag: Squamous cell carcinoma antigen

## References

[1] Ajani JA, Winter KA, Gunderson LL (2008). Fluorouracil, mitomycin, and radiotherapy vs fluorouracil, cisplatin, and radiotherapy for carcinoma of the anal canal: a randomized controlled trial. JAMA.

[2] Ajani JA, Winter KA, Gunderson LL, Pedersen J, Benson AB, Thomas CR, Mayer RJ, Haddock MG, Rich TA, Willett CG (2010). Prognostic factors derived from a prospective database dictate clinical biology of anal cancer: the intergroup trial (RTOG 98-11). Cancer.

[3] Barak V, Goike H, Panaretakis KW, Einarsson R (2004). Clinical utility of cytokeratins as tumor markers. Clin Biochem.

[4] Barak V, Meirovitz A, Leibovici V, Rachmut J, Peretz T, Eliashar R, Gross M (2015). The diagnostic and prognostic value of tumor markers (CEA, SCC, CYFRA 21-1, TPS) in head and neck cancer patients. Anticancer Res.

[5] Correa JHS, Castro LS, Kesley R, Dias JA, Jesus JP, Olivatto LO, Martins IO, Lopasso FP (2013). Salvage abdominoperineal resection for anal cancer following chemoradiation: a proposed scoring system for predicting postoperative survival. J Surg Oncol.

[6] Edge S, Byrd D, Compton CC, Fritz A, Greene F, Trotti A (2010). Anus. AJCC Cancer staging manual.

[7] Giovanella L, Ceriani L, Giardina G, Bardelli D, Tanzi F, Garancini S (2002). Serum cytokeratin fragment 21.1 (CYFRA 21.1) as tumour marker for breast cancer: comparison with carbohydrate antigen 15.3 (CA 15.3) and carcinoembryonic antigen (CEA). Clin Chem Lab Med.

[8] Indinnimeo M, Reale MG, Cicchini C, Stazi A, Fiori E, Izzo P (1997). CEA, TPA, CA 19-9, SCC and CYFRA at diagnosis and in the follow-up of anal canal tumors. Int Surg.

[9] Kuang LI, Song WJ, Qing HM, Yan S, Song FL (2015). CYFRA21-1 levels could be a biomarker for bladder cancer: a meta-analysis. Genet Mol Res.

[10] Mariani P, Ghanneme A, De la Rochefordière A, Girodet J, Falcou MC, Salmon RJ (2008). Abdominoperineal resection for anal cancer. Dis Colon Rectum.

[11] Mitchell SE, Mendenhall WM, Zlotecki RA, Carroll RR (2001). Squamous cell carcinoma of the anal canal. Int J Radiat Oncol.

[12] Molina R, Agusti C, Filella X, Jo J, Joseph J, Giménez N, Ballesta AM (1994). Study of a new tumor marker, CYFRA 21-1, in malignant and nonmalignant diseases. Tumour Biol.

[13] Molina R, Agusti C, Mañe JM, Filella X, Jo J, Joseph J, Giménez N, Estapé J, Ballesta AM (1994). CYFRA 21-1 in lung cancer: comparison with CEA, CA 125, SCC and NSE serum levels. Int J Biol Markers.

[14] Moureau-Zabotto L, Vendrely V, Abramowitz L, Borg C, Francois E, Goere D, Huguet F, Peiffert D, Siproudhis L, Ducreux M, Bouché O (2017). Anal cancer: French intergroup clinical practice guidelines for diagnosis, treatment and follow-up (SNFGE, FFCD, GERCOR, UNICANCER, SFCD, SFED, SFRO, SNFCP). Dig Liver Dis.

[15] Nakamura T, Ide H, Eguchi R, Hayashi K, Takasaki K, Watanabe S (2017). CYFRA 21-1 as a tumor marker for squamous cell carcinoma of the esophagus. Dis Esophagus.

[16] Nakata B, Chung YS, Kato Y, Ogawa M, Ogawa Y, Inui A, Maeda K, Sawada T, Sowa M (1996). Clinical significance of serum CYFRA 21-1 in gastric cancer. Br J Cancer.

[17] Nakata B, Ogawa Y, Ishikawa T, Ikeda K, Kato Y, Nishino H, Hirakawa K (2000). Serum CYFRA 21-1 is one of the most reliable tumor markers for breast carcinoma. Cancer.

[18] Nakata B, Takashima T, Ogawa Y, Ishikawa T, Hirakawa K (2004). Serum CYFRA 21-1 (cytokeratin-19 fragments) is a useful tumour marker for detecting disease relapse and assessing treatment efficacy in breast cancer. Br J Cancer.

[19] National Cancer Institute (2016). Surveillance, epidemiology, and end results program. SEER stat fact sheets: anal Cancer.

[20] Piao X, Kong T-W, Chang S-J, Paek J, Chun M, Ryu H-S (2015). Pretreatment serum CYFRA 21-1 level correlates significantly with survival of cervical cancer patients: a multivariate analysis of 506 cases. Gynecol Oncol.

[21] Pujol JL, Grenier J, Daurès JP, Daver A, Pujol H, Michel FB (1993). Serum fragment of cytokeratin subunit 19 measured by CYFRA 21-1 immunoradiometric assay as a marker of lung cancer. Cancer Res.

[22] Ryan DP, Compton CC, Mayer RJ (2000). Carcinoma of the Anal Canal. N Engl J Med.

[23] Wagner J-P, Mahe MA, Romestaing P, Rocher FP, Berger C, Trillet-Lenoir V, Gerard J-P (1994). Radiation therapy in the conservative treatment of carcinoma of the anal canal. Int J Radiat Oncol Biol Phys.

[24] Wang L, Wang D, Zheng G, Yang Y, Du L, Dong Z, Zhang X, Wang C (2016). Clinical evaluation and therapeutic monitoring value of serum tumor markers in lung cancer. Int J Biol Markers.

[25] Williams M, Swampillai A, Osborne M, Mawdsley S, Hughes R, Harrison M, Harvey R, Glynne-Jones R, Mount Vernon Colorectal Cancer Network (2013). Squamous cell carcinoma antigen: a potentially useful prognostic marker in squamous cell carcinoma of the anal canal and margin. Cancer.

[26] Yuan C, Yang K, Tang H, Chen D (2016). Diagnostic values of serum tumor markers Cyfra 21-1, SCC ag, ferritin, CEA, CA 19-9, and AFP in oral/oropharyngeal squamous cell carcinoma. OncoTargets Ther.

[27] Zhang Z-H, Han Y-W, Liang H, Wang L-M (2015). Prognostic value of serum CYFRA21-1 and CEA for non-small-cell lung cancer. Cancer Med.

